# *Chrysanthemum morifolium* Flower Extract Ameliorates Obesity-Induced Inflammation and Increases the Muscle Mitochondria Content and AMPK/SIRT1 Activities in Obese Rats

**DOI:** 10.3390/nu13103660

**Published:** 2021-10-19

**Authors:** Yoonjin Lee, Jaerin Lee, Mak-Soon Lee, Eugene Chang, Yangha Kim

**Affiliations:** 1 Department of Nutritional Science and Food Management, Ewha Womans University, Seoul 03760, Korea; inuyasha_yj@naver.com (Y.L.); dnflwoflssl@naver.com (J.L.); troph@hanmail.net (M.-S.L.); 2 Department of Food and Nutrition, Gangneung-Wonju National University, Gangneung-si 25457, Korea; echang@gwnu.ac.kr; 3 Graduate Program in System Health Science and Engineering, Ewha Womans University, Seoul 03760, Korea

**Keywords:** *Chrysanthemum morifolium*, obesity, inflammation, muscle mitochondria, AMPK, SIRT1

## Abstract

Decreased energy expenditure and chronically positive energy balance contribute to the prevalence of obesity and associated metabolic dysfunctions, such as dyslipidemia, hepatic fat accumulation, inflammation, and muscle mitochondrial defects. We investigated the effects of *Chrysanthemum morifolium* Ramat flower extract (CE) on obesity-induced inflammation and muscle mitochondria changes. Sprague–Dawley rats were randomly divided into four groups and fed either a normal diet, 45% high-fat diet (HF), HF containing 0.2% CE, or 0.4% CE for 13 weeks. CE alleviated HF-increased adipose tissue mass and size, dyslipidemia, hepatic fat deposition, and systematic inflammation, and increased energy expenditure. CE significantly decreased gene expression involved in adipogenesis, pro-inflammation, and the M1 macrophage phenotype, as well as glycerol-3-phosphate dehydrogenase (GPDH) and nuclear factor-kappa B (NF-kB) activities in epididymal adipose tissue. Moreover, CE supplementation improved hepatic fat accumulation and modulated gene expression related to fat synthesis and oxidation with an increase in adenosine monophosphate-activated protein kinase (AMPK) activity in the liver. Furthermore, CE increased muscle mitochondrial size, mitochondrial DNA (mtDNA) content, and gene expression related to mitochondrial biogenesis and function, including sirtuin 1 (SIRT1), peroxisome proliferator-activated receptor γ coactivator-1α (PGC-1α), and PGC-1α-target genes, along with AMPK-SIRT1 activities in the skeletal muscle. These results suggest that CE attenuates obesity-associated inflammation by modulating the muscle AMPK-SIRT1 pathway.

## 1. Introduction

Obesity results in a chronic energy imbalance between energy intake and energy expenditure. Excess energy intake over energy expenditure results in fat accumulation in white adipose tissue, which is a main feature of obesity [1,2]. Reduced energy expenditure is a critical factor in metabolic regulation in the development of obesity [3,4]. Enlarged adipose tissue resulting from adipocyte hypertrophy and hyperplasia promotes the release of free fatty acids into the circulation and the secretion of pro-inflammatory cytokines from adipose tissue, all of which trigger inflammatory responses within metabolic tissues, such as adipose tissue, the liver, and skeletal muscle [5,6,7]. Obesity-induced chronic inflammation contributes to the development of metabolic disorders, such as dyslipidemia, non-alcoholic fatty liver (NAFLD), type 2 diabetes, hypertension, and cardiovascular disease [8]. Thus, an understanding of the molecular mechanisms of adipose tissue during the development of obesity is critical for the prevention and treatment of obesity and related metabolic dysfunction.

Accumulating evidence demonstrates the pivotal roles played by adenosine monophosphate-activated protein kinase (AMPK) and sirtulin 1 (SIRT1) in energy balance and inflammatory response [9]. In metabolic tissues, such the liver, muscle, and adipose tissue, activation of the AMPK-SIRT1 pathway maintains energy homeostasis by promoting fatty acid oxidation and repressing lipogenesis [10]. Excessive fat accumulation inhibits AMPK/SIRT1 activation in obese rodents, which aggravates obesity-associated metabolic complications [11,12]. In particular, AMPK activation decreases hepatic triglyceride (TG) and increases fatty oxidation by modulating expressions of lipogenic transcription factor, acetyl Co-A carboxylase (ACC), sterol regulatory element binding protein-1c (SREBP-1c) and nuclear receptor, as well as peroxisome proliferator-activated receptor-γ (PPAR-γ) coactivator-1α (PGC-1α). AMPK activity plays a pivotal role in regulating fatty acid metabolism via mitochondrial biogenesis [13,14]. In addition, SIRT1 deficiency exacerbates obesity and inflammation in the adipose tissue of obese mice with suppressed AMPK activity [15]. In relation to AMPK stimulation, SIRT1 is required for muscle mitochondrial function and mitochondrial biogenesis [9]. Skeletal muscle from obese human subjects displays mitochondrial changes and dysfunction described as decreased mitochondrial size and its oxidation capacity [16,17]. Given the close association between obesity and mitochondrial changes in skeletal muscle, it is important to improve obesity-decreased mitochondrial activity for the prevention and/or treatment of obesity and its associated comorbidities. 

*Chrysanthemum morifolium*, a perennial plant in the family Asteraceae, has been used as an alternative medicine with anti-diabetes, anti-oxidant, and anti-tumor activities [18,19,20]. Accumulating evidence demonstrates that apigenin, chlorogenic acid, and luteolin, major bioactive components found in *Chrysanthemum morifolium,* have beneficial effects on hepatic steatosis, hyperlipidemia, insulin resistance, inflammation, and obesity [21,22,23,24,25]. With regard to obesity, only one in vitro study suggests anti-adipogenic effects of *Chrysanthemum morifolium* Ramat flower [26]. In the current study, we investigated the molecular mechanism by which hot water extract of *Chrysanthemum morifolium* Ramat flower (CE) ameliorates high fat diet-induced obesity-related inflammation and skeletal muscle mitochondrial changes in obese rats. 

## 2. Materials and Methods

### 2.1. Preparation of Chrysanthemum morifolium Flower Ramat Extract (CE)

Among the edible *Chrysanthemum morifolium* Ramat, classified into “Bozhu”, “Chuju”, “Gongju”, “Hangzhou” and “Hwaju” varieties, in the present study, we used “Hangzhou” grown in Lanxi, China, from September to November [26]. CE (KL-CTEP009-001) was supplied by Amway Korea (Seoul, Korea) and Amway China R&D (Shanghai, China). *Chrysanthemum morifolium* flowers were extracted in two steps. First, *Chrysanthemum morifolium* flowers were extracted with 30 Bed volume (BV) pure water at a temperature higher than 95 °C for 50 to 60 min. Second, *Chrysanthemum morifolium* flowers were extracted with 8 BV pure water at a temperature higher than 80 °C for 5 min. Then, the fractions were cooled below 25 °C, filtered, and spray-dried into a powder. As described in our previous study [26], CE contained 567.40 ± 3.65, 533.73 ± 3.38, 671.76 ± 3.58, 329.41 ± 2.20, 101.12 ± 0.69, and 427.21 ± 2.28 mg of luteolin-7-O-glucoside, luteolin-7-O-glucuronide, apigenin-7-O-glucoside, chlorogenic acid, 1,5-dicaffeoylquinic acid, and 3,5-dicaffeoylquinic acid per 100 g, respectively.

### 2.2. Animals and Diets

Three-week-old male Sprague–Dawley rats obtained from Doo-Yeol Biotech (Seoul, Korea) were individually housed under conditions of controlled temperature (22 °C), humidity (55%) and lighting (12 h-light and dark cycle). After 1 week of acclimatization to ad libitum water and a normal chow diet (Harlan 2018S rodent diet, Harlan US), rats were randomly assigned into each of following 4 groups (n = 9/group): a normal diet (NOR), 45% high-fat diet (HF), HF supplemented with 0.2% CE (CEL) and 0.4% CE (CEH) as described in Table S1. During the experiment, body weight and food intake were measured weekly. At the end of the 13th week of CE supplementation, all rats were euthanized with a mixture of Zoletil 50 (Virbac Laboratories, France) and Rompun (Bayer Korea, Korea) after 12 h overnight fasting. Blood and tissues were collected and stored at −70 °C for further analysis. All animal experimental procedures were approved by Institutional Animal Care and Use Committee of Ewha Womans University (IACUC No. 19-007).

### 2.3. Measurement of Metabolic Rate

Each rat was placed into an individual metabolic chamber (Oxylet; Panlab, Cornella, Spain) at 25 °C with free access to food and water. O_2_ and CO_2_ analyzers were calibrated with highly purified gas. Oxygen consumption and carbon dioxide production were recorded at 3 min intervals using a computer-assisted data acquisition program (Chart 5.2; AD Instrument, Sydney, Australia) over 12 h (from 6 pm to 6 am). Energy expenditure was calculated according to the following formula: Energy expenditure (kcal/day/kg of body weight) = VO_2_ × 1.44 × [3.815 + (1.232 × VO_2_/VCO_2_)].

### 2.4. Serum Metabolilc Parameters

Serum total cholesterol (TC), triglyceride (TG), and high-density lipoprotein cholesterol (HDL-C) concentrations were measured using commercial kits based on enzymatic colorimetric methods (Embiel Co., Gyeonggi-do, South Korea). The enzymatic kit used to determine serum non-esterified free fatty acids (NFFAs) level was obtained from FUJIFILM Wako Pure Chemicals Co. (Osaka, Japan). The formula of Friedewald [27]; LDL-Cholesterol = Total cholesterol − HDL-C − (TG/5) was employed to calculate serum low- density lipoprotein cholesterol (LDL-C). Serum alanine transaminase (ALT) and aspartate aminotransferase (AST) were measured by enzymatic colorimetric method using commercial kits (Asan pharmaceutical Co., Ltd., Seoul, Korea). Serum nitric oxide (NO) levels were measured using a Griess reagent kit (Invitrogen, Carlsbad, CA, USA) in accordance with the manufacturer’s instruction.

### 2.5. Serum Nitric Oxide (NO) Measurement

Serum NO production was determined by serum nitrite concentration using a commercial kit (Griess reagent kit for nitrate determination; Thermo Scientific, Waltham, MA, USA). Nitrite analysis was based on the nitrosation of sulfanilic acid under acid conditions to form the diazonium salt of sulfanilic acid, which then couples with 1-naphthylamine to form a bright pink azo dye. The absorbance was measured at 548 nm by a spectrophotometric microplate reader (Thermo Scientific). The nitrite concentration was determined using sodium nitrite as a standard and was presented as fold-difference compared to the HF group.

### 2.6. Hepatic Lipid Contents

Hepatic lipids were extracted according to a modified Bligh and Dyer procedure [28]. In brief, 0.5 g of liver tissue was homogenized with 1.5 mL of 0.9% saline. After adding 7.5 mL of chloroform:methanol solution (1:2, *v*/*v*), the mixture was allowed to stand for about 1 h to separate the layers and then 2.5 mL of chloroform was added. Then, the mixture was centrifuged at 3000 rpm for 20 min. The bottom layer was collected, dried, resuspended in n-hexane: isopropanol solution (3:2, *v*/*v*), and then stored at −30 °C until hepatic lipid analysis. TC and TG contents in the liver tissue were measured using enzymatic method kits, as described above.

### 2.7. Hematoxylin and Eosin (H&E) Staining of Adpose Tissue and Liver

Epididymal WAT (eWAT) and liver were fixed using 10% formalin solution for 24 h, embedded into paraffin block, sliced into five-micrometer thickness, and stained for histological analysis. Hematoxylin and eosin (H&E) staining was performed according to the standard procedure. Digital images of H&E-stained tissue sections were observed with a microscope (Olympus, Tokyo, Japan) at 400X magnification and adipocyte size analysis was performed using Image J program (National Institutes of Health, Bethesda, MD, USA). The average adipocyte size was expressed as the average cross-sectional area per cell (1 × 10^2^ μm^2^/cell).

### 2.8. Immunohistochemistry Analysis of Adipose Tissue

eWAT paraffin sections were deparaffinized, rehydrated, incubated with a peroxide blocking buffer for 10 min, and then incubated with rabbit polyclonal anti-F4/80 (EGF-like module-containing, mucin-like, hormone receptor-like 1) (C2C3) antibody solution (GeneTex Inc., Irvine, CA, USA). The immunoreaction was detected with a Polink-2 Plus HRP anti-rat DAB detection kit (Golden Bridge International Inc., Irvine, CA, USA), revealed by a Polink-2 Plus HRP anti-rat 3, 3′-diaminobenzidine tetrahydrochloride (DAB) detection kit (Golden Bridge International Inc., Irvine, CA, USA), and counterstained with Mayer hematoxylin (ScyTek, Logan, UT, USA).

### 2.9. Transmission Electron Microscope Analysis of Skeletal Muscle

Skeletal muscle was pre-fixed with 2% glutaraldehyde buffered by 0.1 M phosphate for 2 h (pH 7.4) and post-fixed with 1% osmium tetroxide buffered by 0.1M PBS for 1 h (pH 7.4), gradually dehydrated with ethanol, embedded in epoxy-resin (Epon 812), sliced into a 1 μm thickness with an ultramicrotome (Reicher-Jung, North Chicago, IL, USA) and stained with 1% toluidine blue. Ultra-thin sections of about 60 to 70 nm were cut and observed using an H-7650 transmission microscope (Hitachi, Tokyo, Japan) at a magnification of 20,000X.

### 2.10. RNA Isolation and Real-Time Quantitative Polymerase Chain Reaction (RT-qPCR)

RiboEx Total RNA reagent (Geneall biotechnology, Seoul, Korea) was used for RNA isolation from liver, muscle, and adipose tissue. cDNA was synthesized using Moloney Murine Leukemia Virus (MMLV) reverse transcriptase kit (Bioneer, Daejeon, Korea). The Rotor-Gene 3000 (Corbett Research, Sydney, Australia) was utilized to conduct quantitative polymerase chain reaction (qPCR) by using the AccuPower 2X Greenstar qPCR Master Mix (Bioneer, Daejeon, Korea). Gene expression was normalized using β-actin, a housekeeping gene and expressed as fold change of the HF group followed by the 2 ^− ∆∆Ct^ method [29]. Primer sequences used for this study are listed in Table S2.

### 2.11. Glycerol-3-Phosphate Dehydrogenase (GPDH) Activity of Adipose Tissue

The homogenate was prepared by adding 0.1 g of eWAT to 200 uL of enzyme extraction buffer. Glycerol-3-phosphate dehydrogenase (GPDH) activity in eWAT was determined by measuring a colorimetric product with absorbance at 340 nm using a commercial kit (Takara, Kyoto, Japan) according to manufacturer’s instructions. GPDH activity was normalized to protein expression which was assessed using a BCA protein assay kit (Thermo Scientific) and expressed as a percentage compared to the HF group.

### 2.12. Nuclear Factor-Kappa B (NF-kB) Measurement of Adipose Tissue

The nuclear fraction was extracted from eWAT using a Nuclear Extraction kit (Abcam, Cambridge, UK). Using a Phospho-NF-kB p65 (Ser536) ELISA kit (RayBiotech Inc., Norcross, GA, USA), eWAT NF-kB level was determined by measuring the phosphorylation of Ser536 on NF-kB with anti-phospho-NF-kB p65 (Ser536) antibody and HRP-conjugated anti-rabbit IgG. Addition of tetramethylbenzidine (TMB) substrate solution develops color in proportion to the amount of NF-kB p65 (Ser536) bound. After reading the absorbance at 450 nm, the values were normalized to total protein of eWAT, which was determined using a BCA protein assay kit (Thermo Scientific) and expressed relatively as fold change of the HF group.

### 2.13. AMPK Activities in the Liver and Skeletal Muscle Tissues

A single-site, semiquantitative immunoassay method was employed to measure hepatic or muscle AMPK activity using a CycLex AMPK kinase assay kit (MBL Life Science, Nagano, Japan) according to the manufacturer’s recommendations. Briefly, AMPK activity was determined by using anti-phospho-mouse IRS-1 S789 monoclonal antibody (AS-4C4), peroxidase coupled anti-mouse IgG antibody to liver homogenates. The absorbance was detected at 450 nm using a plate reader (Thermo Scientific). AMPK activity was normalized to protein concentration as determined by a BCA protein assay kit (Thermo Scientific) and expressed as the fold change compared to the HF group.

### 2.14. Mitochondrial DNA (mtDNA) Levels of Skeletal Muscle

Total DNA was extracted from skeletal muscle using a Gentra Puregene DNA isolation kit (Qiagen, Valencia, CA, USA) according to the manufacturer’s instruction. mtDNA content was measured using RT-qPCR and calculated by the mitochondrial gene (COX1, subunit 1 of cytochrome oxidase) and the nuclear gene (GAPDH, glyceraldehyde 3-phosphate dehydrogenase).

### 2.15. SIRT1 Activity of Skeletal Muscle

A SIRT1 activity assay kit (Abcam) was used to measure SIRT1 activity in the skeletal muscle. Extracted nuclear fraction using the commercial nuclear extract kit (Abcam) without adding protease/peptidase inhibitors was incubated with fluoro-substrate peptide, NAD, and developer. Fluorescence intensity was measured at 340 nm (excitation) and 460 nm (emission) using a microplate reader (Thermo Scientific). SIRT1 activity was normalized to their respective protein concentrations and expressed as the fold change compared to the HF group.

### 2.16. Statistical Analysis

Statistical analyses were conducted with SPSS software (SPSS Inc., Chicago, IL, USA) version 20. Data were expressed as the mean ± standard error of the mean (SEM). Significant differences among the groups were determined by using one-tailed Student’s t-test or one-way analysis of variance (ANOVA) with Tukey’s multiple comparison tests. *p*-values less than 0.05 were considered to be statistically significant.

## 3. Results

### 3.1. Effect of CE on Body Weight Gain and Energy Expenditure

**Figure 1:**
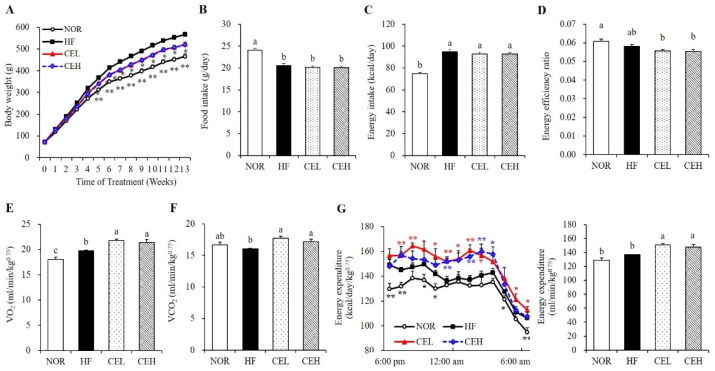
Effect of CE on body weight (**A**), food intake (**B**), energy intake (**C**), energy efficiency ratio (**D**), VO_2_ (**E**) and VCO_2_ consumption (**F**), and energy expenditure (**G**) in obese rats. Values are presented as the mean ± SEM (*n* = 9). Bars with different letters (a,b,c) are significantly different at *p* < 0.05. * *p* < 0.05; ** *p* < 0.01 compared to the HF group. NOR, normal diet; HF, 45% high fat diet; CEL, HF with 0.2% CE; CEH, HF with 0.4% CE. Energy efficiency = body weight gain (g/day)/energy intake (g/day).

After 13 weeks of experiments, final body weights of CEL and CEH were significantly decreased by 7.86% and 8.32%, respectively, compared with the HF group (Figure 1A). Although HF supplemented with CE significantly decreased body weight gains by 8.96% (CEL) and 9.60% (CEH), there were no significant differences in food intake, energy intake, or energy efficiency (Figure 1B–D). To evaluate the effect of CE on energy expenditure, we measured metabolic rates using indirect calorimetry. As shown in Figure 1E, oxygen consumption in the CEL and CEH groups significantly increased by 10.33% and 8.30% respectively, compared with the HF group (Figure 1E, *p* < 0.05). Carbon dioxide production in the CEL and CEH groups was significantly higher by 10.34% and 6.92% than the HF group, respectively (Figure 1F, *p* < 0.05). In addition, the levels of energy expenditure in the CEL and CEH group were significantly higher than the HF group by 10.32% and 7.98%, respectively (Figure 1G, *p* < 0.05). These observations suggest the favorable effect of CE on obesity might be in part related to increased energy expenditure.

### 3.2. CE Prevents Obesity-Induced Dyslipidemia

Serum lipid concentrations are shown in Table 1. Serum TG, TC, and LDL levels of the CEL group were significantly reduced by 13.85%, 19.92%, and 37.61% respectively, compared to the HF group (*p* < 0.05). Furthermore, serum TG, TC, and LDL levels of CEH group were significantly decreased by 16.92%, 24.36%, and 50.46%, respectively (*p* < 0.05). Serum NEFA contents in the CEL and CEH groups were significantly reduced by 17.55% and 17.26%, respectively, compared to the HF group (*p* < 0.05). However, 0.2% and 0.4% CE supplementation did not change serum AST and ALT levels, which are indicators of liver toxicity.

### 3.3. CE Inhibits Adipocyte Hypertrophy and Adipogenic Gene Expression in eWAT

**Figure 2:**
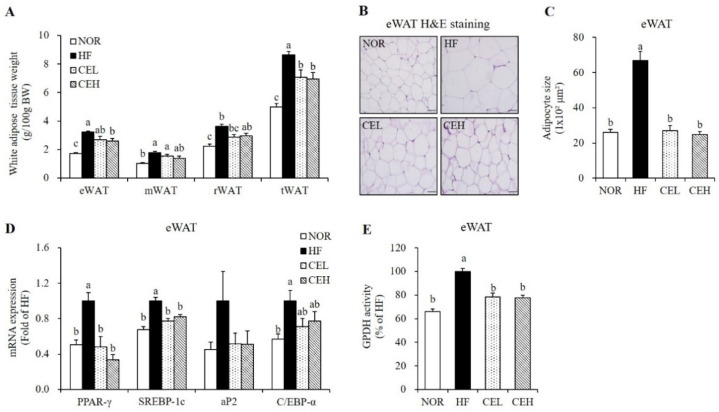
Effect of CE on adipogenesis and lipid accumulation in eWAT. (**A**) White adipose tissue weights. (**B**) Representative H&E-stained eWAT sections (magnification, 400×; Scale bar, 50 µm). (**C**) Average adipocyte size. (**D**) Gene expression of PPAR-γ, SREBP-1c, aP2, and C/EBP-α was measured by RT-qPCR and normalized to β-actin. Data are expressed as the fold change compared to the HF group. (**E**) Glycerol-3-phosphate dehydrogenase (GPDH) activity. Data are presented as a percentage versus the HF group. Values are presented as the mean ± SEM (*n* = 9). Bars with different letters (a,b,c) are significantly different (*p* < 0.05). NOR, normal diet; HF, 45% high fat diet; CEL, HF with 0.2% CE; CEH, HF with 0.4% CE; eWAT, epididymal white adipose tissue; mWAT, mesenteric white adipose tissue; rWAT; retroperitoneal white adipose tissue; tWAT, total white adipose tissue.

As shown in Figure 2, excessive energy consumption with HF significantly increased epididymal (eWAT), mesenteric (mWAT), retroperitoneal (rWAT), and total white adipose tissue (tWAT) weights by 85.47%, 74.84%, 62.13%, and 72.86% respectively, compared to the NOR diet (*p* < 0.05). CE supplementation tended to reduce mWAT and rWAT weights in a dose-dependent manner but the difference was not significant. eWAT weight was significantly decreased by CE in a dose dependent manner, started at CEL (*p* < 0.05). tWAT masses in the CEL and CEH groups was significantly lower than in the HF group by 18.15% and 19.54%, respectively (Figure 2A, *p* < 0.05). Consistent with eWAT mass, average adipocyte size of eWAT appeared smaller in obese rats fed with CE (Figure 2B). As shown in Figure 2C, the average adipocyte size of the HF group was larger than that of the NOR group. CE supplementation (CEL and CEH groups) significantly decreased HF-induced adipocyte size by 59.57% and 62.97% respectively, compared to the HF group (Figure 2C, *p* < 0.05). mRNA levels of PPAR-γ, SREBP-1c, adipocyte fatty acid binding protein2 (aP2), and CCAAT/enhancer binding protein-α (C/EBP-α) involved in adipogenesis was upregulated by HF diet, compared to the NOR diet. CE supplementation significantly decreased PPAR-γ and SREBP-1c mRNA levels by at least 51.78% and 17.64%, respectively (*p* < 0.05). The mRNA expression of aP2 and C/EBP-α was lower in eWAT of CE-fed obese rats but the difference was not significant (Figure 2D). Increased PPAR-γ mRNA expression is associated with increased GPDH activity, which provides the glycerol 3-phosphate required for the TG synthesis [30]. CE significantly suppressed GPDH activity compared to the HF group (Figure 2E) (*p* < 0.05).

### 3.4. CE Suppresses Obesity-Associated Adipose Tissue Macrophage Infiltration and Systematic Inflammation

**Figure 3:**
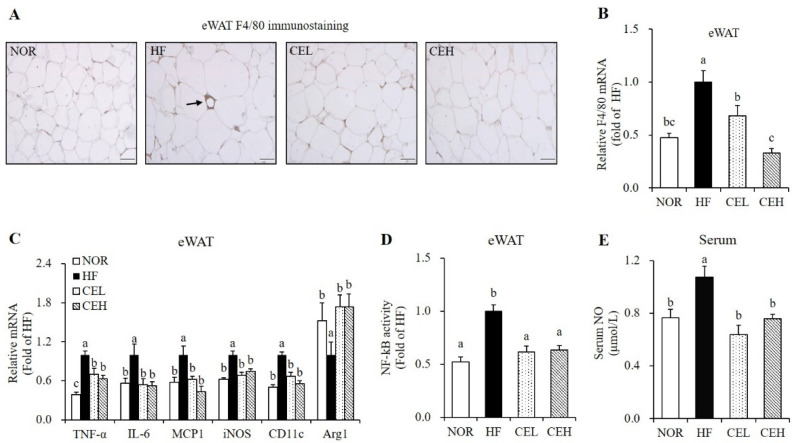
Influence of CE on adipose tissue macrophage infiltration and polarization and serum nitric oxide concentration. (**A**) Representative images of F4/80 immunostaining in epididymal white adipose tissue (magnification, 400×; scale bar, 50 µm). The arrow indicates a crown like structure (CLS). (**B**) F4/80 gene expression, (**C**) mRNA levels involved in inflammation and M1/2 macrophage markers were measured by RT-qPCR and normalized to β-actin. Data are expressed as the fold change as compared to the HF group. (**D**) Nuclear phospho-NF-κB p65 level was determined by a Phospho-NF-kB p65 (Ser536) ELISA kit, normalized to protein concentration, and expressed as the fold change to the HF group. (**E**) Serum nitric oxide (NO) concentration was determined using a Griess reagent kit and expressed as the fold change compared to the HF. Values are expressed as mean ± SEM (*n* = 9). Bars with different letters (a, b, c) show significant differences (*p* < 0.05). NOR, normal diet; HF, 45% high fat diet; CEL, HF with 0.2% CE; CEH, HF with 0.4% CE; eWAT, epididymal white adipose tissue.

Next, we investigated whether CE attenuated HF-induced adipose tissue macrophage (ATM) infiltration and M1 polarization by eWAT immunostaining with F4/80 antibody. As shown in Figure 3A, eWAT in the HF group exhibits adipocyte death and macrophage infiltration as evidenced by crown-like structures (CLSs), but not in the NOR group and CE-supplemented groups. This suggests the favorable effect of CE on obesity-related ATM accumulation. F4/80 mRNA expression in the CEL and CEH groups were significantly decreased by 32.00% and 34.75%, respectively, compared to the HF group (Figure 3B) (*p* < 0.05). In addition, eWAT mRNA levels involved in inflammation and ATM polarization were measured by RT-qPCR (Figure 3C). CE supplementation significantly down-regulated HF-increased TNF-α, IL-6, and MCP1 gene expression in eWAT (*p* < 0.05). In addition, M1 macrophage marker, CD11c (known as integrin alpha X) and inducible nitric oxide synthase (iNOS) mRNA levels were suppressed by CE supplement (*p* < 0.05). On the other hand, gene expression of arginase1 (Arg1), a M2 macrophage marker was significantly increased by about 1.73-folds in both CEL and CEH groups compared to the HF group (Figure 3C) (*p* < 0.05). Moreover, NF-kB activities in the CEL and CEH groups were significantly decreased by 0.39-fold and 0.36-fold respectively, compared with the HF group (Figure 3D) (*p* < 0.05). Next, we measured serum NO level to investigate the effect of CE supplementation on systematic inflammation in obese rats. Serum NO concentrations, a molecule synthesized by iNOS, was measured to investigate the effects of CE on systemic inflammation. CEL and CEH groups significantly reduced serum NO levels by 40.60% and 29.25% respectively, compared to the HF group (Figure 3E) (*p* < 0.05).

### 3.5. CE Ameliorates HF-Induced Hepatic Fat Depostion and Increase Hepatic AMPK Activity

**Figure 4:**
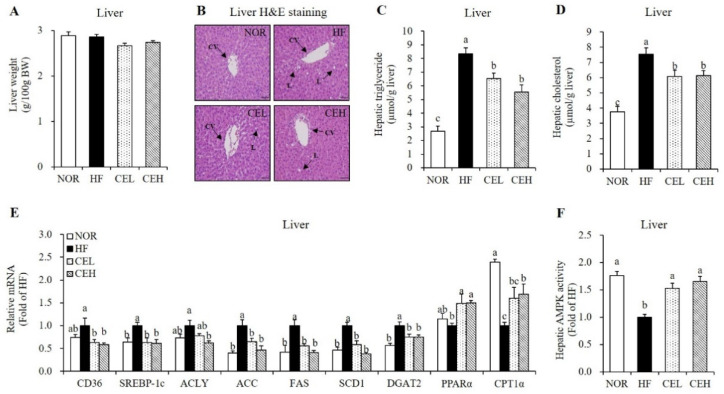
Influence of CE on hepatic fat infiltration and gene expression related to fatty acid uptake and oxidation and lipogenesis. (**A**) Liver tissue weight. (**B**) Representative H&E-stained liver sections (magnification, 400×; Scale bar, 50 µm). Black arrows indicate central vein (CV) and lipid deposition (L). Hepatic TG (**C**) and cholesterol levels (**D**) were expressed as μmol/g liver. (**E**) Hepatic gene expression involved in lipid metabolism were measured by RT-qPCR and normalized to β-actin. Data are expressed as the fold change as compared to the HF group. (**F**) AMPK activity was measured using a CycLex AMPK kinase assay kit, normalized with protein expression, and expressed as the fold change compared to the HF group. Values are expressed as mean ± SEM (*n* = 9). Bars with different letters (a, b, c) show significant differences (*p* < 0.05). NOR, normal diet; HF, 45% high fat diet; CEL, HF with 0.2% CE; CEH, HF with 0.4% CE.

To demonstrate the influence of CE on lipid accumulation in the liver tissue, H&E staining and measurement of hepatic lipid profiles were employed. CE supplementation reduced the HF-induced number and size of hepatic lipid droplet, observed by H&E stained liver section with a tendency to decrease liver weight although the change was not significant (Figure 4A,B). In the CEL and CEH groups, hepatic TG levels were significantly lower by 21.90% and 33.71% respectively, than that of the HF group (Figure 4C, *p* < 0.05). In addition, hepatic TC levels were significantly decreased in the CEL and CEH groups by 19.39% and 18.46% respectively, compared to the HF group (Figure 4D, *p* < 0.05). Hepatic gene expression related to lipogenesis and fatty acid oxidation was analyzed by RT-qPCR (Figure 4E). CE significantly down-regulated HF-induced hepatic lipogenic gene expression such as cluster of differentiation 36 (CD36), SREBP1-c, ATP citrate lyase (ACLY), ACC, fatty acid synthase (FAS), stearoyl-CoA desaturase 1 (SCD1), and diacylglycerol O-acyltransferase 2 (DGAT2) in rats fed a HF diet (*p* < 0.05). Moreover, gene expression involved in fatty acid oxidation such as PPARα and carnitine palmitoyltransferase 1α (CPT1α), were significantly higher in the CE groups than those of the HF group (*p* < 0.05). AMPK, a heterotrimeric protein complex inhibits ACC activation, involved in hepatic lipid synthesis and inflammation [13,14]. AMPK activation was significantly increased by 1.53-fold and 1.65-fold, respectively, in CE supplement groups compared with the HF group (Figure 4F, *p* < 0.05).

### 3.6. CE Stimulates Mitochondrial Gene Expression and AMPK/SIRT1 Activities in the Skeletal Muscle

**Figure 5:**
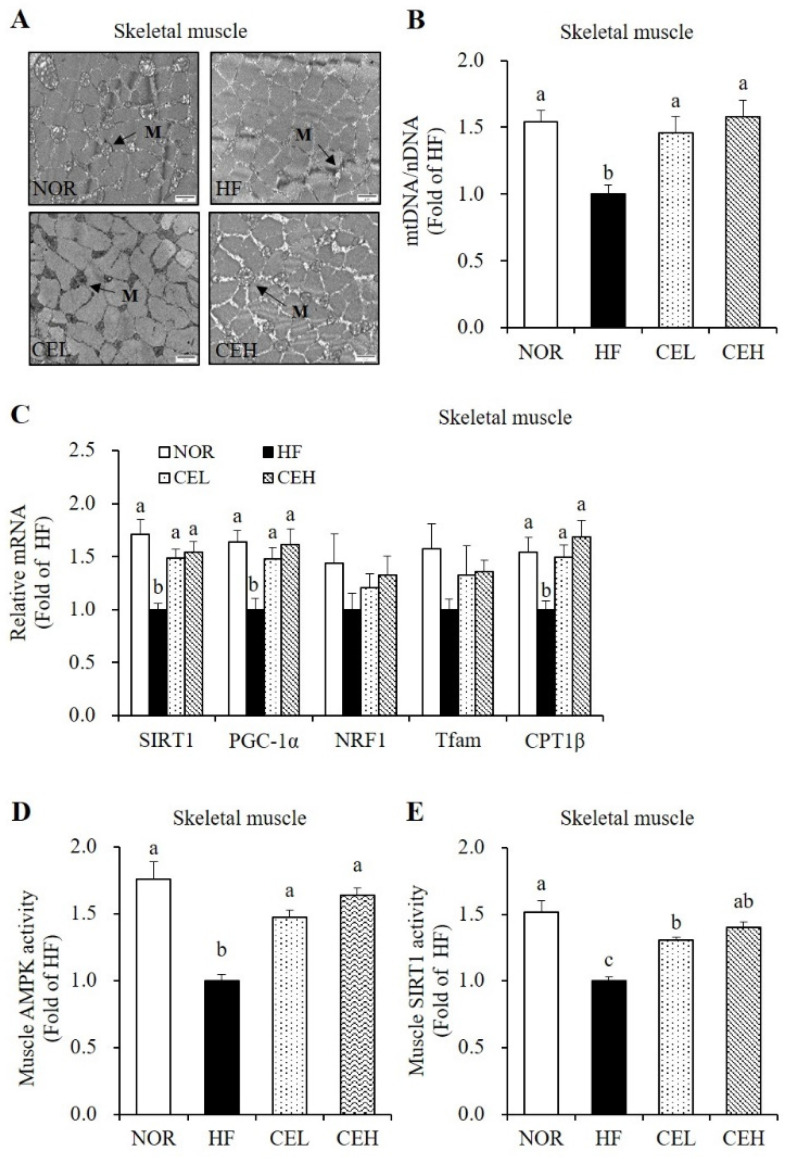
Effect of CE on mitochondrial morphology, mitochondrial DNA (mtDNA) contents, mRNA levels related to mitochondrial biogenesis and function, and AMPK/SIRT1 activities in the skeletal muscle. (**A**) Transmission electron microscopy of skeletal muscle (magnification, 20,000×; Scale bar, 2 µm). M represents the position of mitochondria. (**B**) mtDNA transcripts were determined by RT-qPCR. (**C**) mRNA levels of SIRT1, PGC-1α, NRF1, Tfam, and CPT1β were measured by RT-qPCR and normalized to β-actin. Data are expressed as the fold change as compared to the HF group. (**D**) A CycLex AMPK kinase assay kit was used to measure muscle AMPK activity. Results were normalized to protein concentration and expressed as the fold change of the HF group. (**E**) SIRT1 activity was analyzed by a fluorometric SIRT1 activity assay kit, normalized to protein concentration, and expressed as the fold change compared to the HF group. Values are expressed as means ± SEM (*n* = 9). Bars with different letters (a, b, c) show significant differences (*p* < 0.05). NOR, normal diet; HF, 45% high fat diet; CEL, HF with 0.2% CE; CEH, HF with 0.4% CE.

Next, we determined the beneficial effects of CE on obesity-decreased mitochondrial biogenesis and function in the skeletal muscle. In a TEM analysis, CE supplementation revealed the larger size and number of skeletal muscle mitochondria compared to the HF group (Figure 5A). In addition, CEL and CEH significantly increased HF-decreased mtDNA levels by 1.46- and 1.58-fold, respectively, in a dose-dependent manner (Figure 5B *p* < 0.05). mRNA levels of SIRT1, PGC-1α, nuclear respiratory factor 1 (NRF1), transcription factor A (Tfam), and CPT1β related to mitochondrial biogenesis and function in the skeletal muscle were measured by RT-qPCR. CE supplementation significantly upregulated HF-decreased SIRT1 and PGC-1 α gene expression by about 1.50-fold (*p* < 0.05), but increased tendency of NRF1 and Tfam expression was found by CE without statistical difference (Figure 5C). CPT1β mRNA expression involved in fatty acid oxidation was significantly suppressed by HF diet, which was reversed by CE supplementation (*p* < 0.05). In accordance with SIRT1 mRNA level, CEL and CEH group demonstrated significant increase in muscle SIRT1 activity by 1.31- and 1.40-fold, compared to the HF group (Figure 5D *p* < 0.05). As shown in Figure 5E, HF-decreased AMPK activity was significantly increased by CE supplementation (CEL and CEH groups) by 1.47- and 1.64-fold (*p* < 0.05).

## 4. Discussion

In the present study, we demonstrate that CE supplementation reduces body fat adiposity and obesity-related inflammation in HF-fed obese rats. The two doses of CE used in this study were 0.2% and 0.4%, based on previous studies investigating anti-obesity effect of *Chrysanthemum morifolium* and its major active components, luteolin and chlorogenic acid [21,24,31,32]. In our previous study using ultra-performance liquid chromatography (UPLC) analysis, we found that CE contains 2630.63 ± 15.37 mg bioactive compounds/100 g of powered sample including luteolin-7-O-glucuronide, chlorogenic acid, 1,5-dicaffeoylquinic acid, cynaroside, 3,5-dicaffeoylquinic acid, and apigenin–7–O-glucoside [26]. These doses did not increase further HF-increased serum ALT and AST levels. To evaluate the effects of CE on dyslipidemia, serum lipid profiles were analyzed. A 13-week CE supplementation improved HF-induced FFA, TC, TG, and LDL-C, consistent with a previous study showing favorable effects of 6-week supplementation with *Chrysanthemum* ethanol extract on serum lipid levels [33]. In addition, luteolin, one of the most abundant polyphenols in CE, significantly reduces circulating FFA, TG and TC concentrations [24,32]. Another bioactive component in CE, chlorogenic acid shows hypolipidemic effects via modulating fatty acid metabolism [21]. Therefore, the favorable effect of CE on obesity-related serum lipid abnormalities might be associated with beneficial effects of bioactive components contained in CE.

Chronic energy surplus leads to obesity, characterized by enlarged adipose tissue [2]. In this study, dietary CE supplementation significantly decreased HF-induced body weight, total WAT mass, and eWAT adipocyte cell size without changing food intake. However, VO_2_, VCO_2_, and energy expenditure was significantly higher in CE groups than HF group. Consistent with our results, the ethanol extract of *Chrysanthemum morifolium* significantly increases EE and VO_2_ [34]. Growing evidence illustrates that energy expenditure is pivotal to metabolic control in the development of obesity [3,4]. Therefore, CE was thought to alleviate HF-induced obesity by increasing oxygen consumption and energy expenditure. Unexpectedly, there was no difference in energy expenditure between NOR and HF groups when measuring indirect calorimetry and oxygen consumption over 12 h from 6 pm to 6 am. To fully resolve the molecular mechanism by which CE-increased energy expenditure improves HF-induced obesity, first, further research is warranted to carry out indirect calorimetry for 24 h. Next, we analyzed adipogenic gene expression in eWAT to investigate the effect of CE supplementation on adipocyte size. Consistent with the results of our previous study showing that CE decreased adipocyte differentiation in 3T3-L1 cells [26], CE supplementation significantly reduced adipogenic gene expression such as PPAR-γ, SREBP-1c, and C/EBP-α in eWAT from obese rats. PPAR-γ and C/EBP-α are key transcription factors for adipogenesis [35,36]. SREBP1-c is a lipogenic transcriptional factor for de novo fatty acid synthesis [36]. In a previous study, the water extract of *Chrysanthemum morifolium* reduced PPAR-γ and aP2 protein levels in eWAT from obese diabetic KK-Ay mice [19]. Due to the close association between PPAR-γ expression and GPDH activity for TG synthesis [30], we also investigated GDPH activity in eWAT. In the present study, CE groups decreased eWAT GPDH activity. These results suggest that CE improved adiposity via downregulating adipogenic gene expression and adipogenesis-related enzyme activity with the increment of energy expenditure.

Given a close association between obesity and low grade chronic inflammation [5], we investigated the favorable effect of CE on obese-associated inflammation. In this study, CE-decreased ATM, as determined by CLS in F4/80-immunostained eWAT was observed together with a decrease of F4/80 mRNA level. This finding suggests that CE plays a role in the decrement of obesity-induced adipose inflammation. Adipose tissue inflammation results from increased M1 (pro-inflammatory)/M2 (anti-inflammatory) phenotypic switching with subsequent secretion of adipocyte-derived pro-inflammatory cytokines [37]. In the present study, TNF-α, IL-6, and MCP1 mRNA levels in eWAT were significantly decreased by CE supplementation. NF-κB is a key transcription factor that controls expression of pro-inflammatory cytokines such as TNF-α and IL-6 [38]. In the present study, NF-κB activity was reduced by the CE supplementation, similar to the results of a study illustrating that luteolin attenuates inflammatory responses in adipocytes by NF-κB inhibition [24]. Given the relationship between macrophage polarization and pro-inflammatory cytokine, we next measured mRNA expressions of iNOS and CD11c, known as M1 macrophage markers, and Arg1, a M2 macrophage marker. CE significantly down-regulated HF-increased iNOS and CD11c mRNA levels and increased Arg1 mRNA expression in eWAT from HF-fed obese rats. It was previously reported that luteolin, a major bioactive component found in CE suppressed adipose tissue M1 polarization and mRNA levels of TNF-α, IL-6, and MCP1 of eWAT from HF-fed obese mice [22]. Therefore, we speculate that the favorable effects of CE on ATM transformation and pro-inflammatory cytokine are partially due to the activity of luteolin. In addition, circulating NO produced by iNOS was significantly reduced by CE, implying that CE ameliorates obesity-related systematic inflammation. These results suggest that CE alleviates obesity-induced adipose tissue and systematic inflammation, in part, by inhibiting NF-κB signaling pathways and eWAT mRNA expression related to inflammation.

Chronic energy imbalance contributes to circulating FFA level released from enlarged adipose tissue and subsequently increases hepatic fat synthesis and fat accumulation [7,8]. Increased hepatic fat oxidation has been considered as an effective strategy for the treatment of NAFLD [39]. With the beneficial effect of CE on HF-induced hepatic fat deposition demonstrated in our present study, 0.2% and 0.4% CE in the HF significantly reduced hepatic TC and TG levels with the decrement of hepatic lipid droplets observed by H&E staining. Furthermore, CE supplementation significantly reduced the mRNA expression involved in hepatic lipogenesis, such as CD36, SREBP1-c, ACLY, ACC, FAS, SCD1, and DGAT2. Similar to the present study, the ethanol extract of *Chrysanthemum* flowers attenuated hepatic fat accumulation in Kunming mice fed a high fat milk [33]. In relation to CE-decreased fatty liver, one of the main phenolic compounds in CE, chlorogenic acid significantly decreased hepatic lipid levels with the reduction of hepatic CD36 and FAS mRNA levels in mice fed a HF diet [25]. With respect to de novo lipogenesis in the liver tissue, circulating fatty acids are taken up by CD36 [40]. ACLY converts citrate to acetyl-CoA, which is converted to malonyl-CoA by ACC in the process of de novo fatty acid synthesis [41,42]. FAS generates long chain fatty acids with the use of acetyl-CoA and malonyl-CoA [43]. Growing evidence demonstrates a major role of AMPK in lipid and energy metabolism by inhibiting hepatic lipogenesis and increasing fatty acid oxidation via suppressing ACC activity [13,44]. In the current study, hepatic mRNA levels of PPARα and CPT1α involved in fatty acid oxidation were significantly increased by CE in obese rats. Moreover, CE supplementation significantly increased AMPK activity in the liver tissue. Therefore, our results suggest that CE inhibits hepatic lipid accumulation by activating AMPK and modulating hepatic gene expression involved in fatty acid and TG synthesis and fatty acid oxidation in obese rats.

Skeletal muscle mitochondrial dysfunction including incomplete fatty acid oxidation occurs in a positive energy balance [16,45]. Given close association between muscle mitochondrial coupling efficiency and mitochondrial biogenesis, in this study, HF-decreased mitochondrial size was observed by TEM analysis, which was reversed by the CE. In addition to this observation, CE supplementation significantly increased mtDNA content and mRNA levels of SIRT1, PGC-1α, NRF1, Tfam, and CPT-1β with increased SIRT1 activity in the skeletal muscle from HF-fed obese rats. In addition, HF-decreased AMPK phosphorylation in the skeletal muscle were significantly upregulated by CE supplementation. SIRT1, a NAD-dependent protein deacetylase has emerged as an important regulator of energy metabolism by enhancing energy expenditure and reducing body fat gain [9,15,46]. In the regulation of energy metabolism, SIRT1 is involved in several transcription factors for mitochondrial biogenesis and function. SIRT1 interacts with, deacetylates, and activates PGC-1α, a transcriptional coactivator for NRF1 and Tfam, in accordance with AMPK activity [9]. In mitochondrial matrix, Tfam stimulates mtDNA replication and mitochondrial gene expression [47]. In addition, PGC-1α is involved in mitochondrial oxidative function and the subsequent energy expenditure by promoting target PPARα gene transcription. PPARα stimulates the fatty acid oxidation-related gene, CPT1 [48]. Similar to the beneficial effect of CE on muscle mitochondria in the present study, ethanol extract of *Chrysanthemum zawadskil* Herbich ameliorates dexamethasone-induced muscle atrophy and mitochondrial dysfunction in C57BL/6 mice [49]. In addition, chlorogenic acid, one of the most abundant polyphenols in CE, improves muscle strength, mitochondrial function, and cellular energy metabolism [50]. Thus, we suggest that the favorable mechanism of CE on obesity and associated inflammation might be through the improvement of muscle mitochondrial biogenesis and function with AMPK/SIRT1 activation.

About 40% of body mass is skeletal muscle, which plays a pivotal role in the maintenance of metabolic health in human. In rodent whole-body energy homeostasis, BAT plays more relevant compared to skeletal muscle. Although the dose-dependent effects of CE on muscle mtDNA levels and mitochondrial biogenesis markers were not matched with equivalent dose-response effect of CE on body weight, energy expenditure, or other metabolic markers, this might be one of plausible mechanisms. Still, further investigation is needed to delineate whether CE-increased energy expenditure mitigates obesity by regulating oxidative respiration, peripheral and systemic fatty acid oxidation, and energy homeostasis and mitochondrial biogenesis and function not only in skeletal muscle but also in brown adipose tissue (BAT), WAT, and liver and by inducing BAT activation and WAT browning. Given the link between muscle mitochondrial coupling efficiency and obesity-related metabolic disturbance, it is necessary to measure whole-body expenditure, oxygen consumption, glucose tolerance test, insulin tolerance test, and hyperinsulinemic–euglycemic clamps.

## 5. Conclusions

In the present study, 13 weeks of supplementation with CE ameliorated obesity-induced inflammation in obese rats. CE supplementation attenuated HF-increased adipose tissue mass and inflammation, M1/M2 phenotype switching, and systematic inflammation by modulating gene expression of adipogenesis, pro-inflammation, and M1/M2 markers. The favorable effect of CE on dyslipidemia and NAFLD might be associated with hepatic AMPK activity and mRNA levels related to lipid synthesis and fatty acid oxidation. In addition, CE attenuated obesity-associated decrement in muscle mitochondrial mass and function, partially by modulating AMPK/SIRT1 pathway. Thus, it is assumed that CE might be useful as a nutraceutical to improve obesity-associated inflammation and muscle mitochondrial dysfunction.

## Figures and Tables

**Table 1:** Effects of CE supplementation on serum lipid levels in obese rats.

	NOR	HF	CEL	CEH
NEFA (mEq/L)	0.64 ± 0.03 ^b^	0.77 ± 0.03 ^a^	0.64 ± 0.04 ^b^	0.64 ± 0.03 ^b^
TG (mmol/L)	1.12 ± 0.02 ^b^	1.30 ± 0.06 ^a^	1.12 ± 0.03 ^b^	1.08 ± 0.02 ^b^
TC (mmol/L)	3.74 ± 0.11 ^b^	4.72 ± 0.24 ^a^	3.78 ± 0.10 ^b^	3.57 ± 0.05 ^b^
HDL-C (mmol/L)	2.09 ± 0.04	1.95 ± 0.03	1.91 ± 0.08	2.00 ± 0.04
LDL-C (mmol/L)	1.14 ± 0.13 ^b^	2.18 ± 0.23 ^a^	1.36 ± 0.14 ^b^	1.08 ± 0.08 ^b^
ALT (IU/L)	14.72 ± 0.79	11.84 ± 0.89	11.94 ± 0.72	12.10 ± 0.65
AST (IU/L)	56.59 ± 1.29	55.63 ± 3.42	52.92 ± 1.85	51.85 ± 3.48

Data are presented as the mean ± SEM (*n* = 9). Values with different letters (a,b) show significant difference at *p* < 0.05. NOR, normal diet; HF, 45% high fat diet; CEL, HF with 0.2% CE; CEH, HF with 0.4% CE; NEFA, non-esterified fatty acids; TG, triglyceride; TC, total cholesterol; LDL-C, LDL-cholesterol; HDL-C, HDL-cholesterol; ALT, alanine transaminase; AST, aspartate aminotransferase.

## Data Availability

The data presented in this study are available from the corresponding author upon request. The data are not publicly available due to privacy.

## Supplementary Materials

The following are available online at https://www.mdpi.com/article/10.3390/nu13103660/s1, Table S1: The composition of experimental diets, Table S2: Primers used for real-time quantitative polymerase chain reaction.
