# Supplementary material for: Chrysanthemum morifolium Flower Extract Ameliorates Obesity-Induced Inflammation and Increases the Muscle Mitochondria Content and AMPK/SIRT1 Activities in Obese Rats
Source: Nutrients. 2021 Oct 19;13(10):3660. doi: 10.3390/nu13103660 (PMC8539674; doi:10.3390/nu13103660)
Supplement: Supplementary file 1 [file nutrients-13-03660-s001.zip › nutrients-1371278-supplementary.pdf]

nutrients MDPI

Table S1. The composition of experimental diets.

Component (g / kg diet)	NOR [1]	HF [2]	CEL	CEH
Casein	-	170.73	170.73	170.73
Sucrose	-	121.95	121.95	121.95
Dextrose	-	155.00	155.00	155.00
Corn starch	-	201.71	199.71	197.71
Cellulose	-	60.98	60.98	60.98
Lard	-	229.50	229.50	229.50
Mineral mix [2]	-	42.68	42.68	42.68
Vitamin mix [3]	-	12.20	12.20	12.20
L-cystein	-	2.20	2.20	2.20
Choline bitartrate	-	3.05	3.05	3.05
Chrysanthemum morifolium extract (CE)	-	-	2.0	4.0
Total	-	1000	1000	1000
Energy density (kcal/g)	3.1	4.6	4.6	4.6
Carbohydrates (% as kcal)	58	41.5	41.5	41.5
Protein (% as kcal)	24	13.5	13.5	13.5
Fat (% as kcal)	18	45.0	45.0	45.0

[1] Normal chow diet (Harlan 2018S rodent diet, Harlan US); [2] AIN-93G mineral mix; [3] AIN-93 vitamin mix. NOR, normal diet; HF, 45% high fat diet; CEL, HF with 0.2% *Chrysanthemum morifolium* extract (CE); CEH, HF with 0.4% *Chrysanthemum morifolium* extract (CE).

Copyright: © 2021 by the authors. Licensee MDPI, Basel, Switzerland. This article is an open access article distributed under the terms and conditions of the Creative Commons Attribution (CC BY) license (https://creativecommons.org/licenses/by/4.0/).

Table S2. Primers used for quantitative real time polymerase chain reaction.

Name	GeneBank No.	Primer sequence (5′-3′)
aP2	NM_053365	F: TCACCCCAGATGACAGGAAA<br>R: CATGACACATTCCACCACCA
ACC	NM_022193	F: GAAAAGCGATTCCCATCCGC<br>R: CATTCCATGCAGTGGTCCCT
ACLY	BC100618.1	F: GCCAGGGAGCTGGGTTTAAT<br>R: CACCACCAATGCCCATCTCT
Arg1	NM_017134.3	F: ACATCGGCTTGCGAGATGTG<br>R: GCCAATTCCCAGCTTGTCCA
β-actin	NM_031144	F: GGCACCACACTTTCTACAAT<br>R: AGGTCTCAAACATGATCTGG
CD11c	XM_006230382.3	F: CAGAACCCGTCCACCCAATG<br>R: GATGTCACAGCGGAAGTGCA
C/EBP-α	NM_012524	F: GCCAAGAAGTCGGTGGATAA<br>R: CGGTCATTGTCACTGGTCAA
CD36	NM_031561	F: CCTGTGAGTTGGCAAGAAGC<br>R: AATGAGCCCACAGTTCCGAT
CPT1α	NM_031559	F: TCGGCAGACCTATTTTGCAC<br>R: ATTTGGCGTAGCTGTCGATG
CPT1β	NM_013200	F: TGTACTAGCGAGTCCACGGC<br>R: GGTGTTTTTCGGAGGCTTTC
DGAT2	NM_001012345	F: CGTGTGGCGCTATTTTCGAG<br>R: GGCCTTATGCCAGGGAACTT
FAS	NM_017332	F: GCAGCAGCATGATGTAGCAC<br>R: AGTTGCACACCACAAGGTCA
IL-6	NM_012589	F: ATAGTCCTTCCTACCCCAAC<br>R: TGCCGAGTAGACCTCATAGT
iNOS	NM_012611.3	F: TCCTGCCACCTTGGAGTTCA<br>R: TGGTCACCTCCAGCACAAGA
MCP1	NM_031530	F: ACTCACCTGCTGCTACTCAT<br>R: CTACAGCTTCTTTGGGACAC
NRF1	NM_01100708	F: CTGTGGCTGATGGAGAGGTG<br>R: CACTGTTAAGGGCCATGGTG
PGC1-α	NM_031347	F: GCACCAGAAAACAGCTCCAA<br>R: TTACTGAAGTTGCCATCCCG
PPARα	NM_013196	F: TACCTGTGAACACGATCTGA<br>R: GCTAGTCTTTCCTGCGAGTA
PPARγ	NM_001145366	F: TGTGGGGATAAAGCATCAGG<br>R: CAAGGCACTTCTGAAACCGA
SCD1	NM_139192	F: GTGGCAGGGCAGGAAATAGT<br>R: CAACACCACAAGAAGCCACG
SIRT1	XM_008772947	F: AGGGAACCTCTGCCTCATCT<br>R: GAGGTGTTGGTGGCAACTCT
SREBP-1c	AF286470	F: AGGAGGCCATCTTGTTGCTT<br>R: GTTTTGACCCTTAGGGCAGC
Tfam	NM_0311326	F: TGGGCTTAGAGAAGGAAGCC<br>R: TGCTGACCGAGGTCTTTTTG
TNF-α	NM_012675	F: CCCCTTTATCGTCTACTCCT<br>R: ACTACTTCAGCGTCTCGTGT

aP2, adipocyte protein 2; ACC, acetyl-CoA carboxylase; ACLY, ATP citrate lyase; Arg1, arginase 1; CD11c, cluster of differentiation 11c; C/EBP-α, CCAAT/enhancer-binding protein-α; CD36, cluster of differentiation 36; CPT1, carnitine / palmitoyl-transferase 1; DGAT2, diacylglycerol O-acyltransferase; FAS, fatty acid synthase; IL-6, interleukin-6; iNOS, inducible nitric oxide synthase; MCP1, monocyte chemoattractant protein 1; NRF1, nuclear respiratory factor 1; PGC-1α, peroxisome proliferator-activated receptor gamma coactivator 1α; PPARα, Peroxisome proliferator-activated receptor α; PPARγ, peroxisome proliferator-activated receptor γ; SCD1, stearoyl-CoA desaturase 1; SIRT1, sirtuin 1; SREBP-1c, sterol regulatory element binding protein-1c; Tfam, transcription factor A; TNF-α, tumor necrosis factor-α.
